# Importance of Ethnicity, CYP2B6 and ABCB1 Genotype for Efavirenz Pharmacokinetics and Treatment Outcomes: A Parallel-Group Prospective Cohort Study in Two Sub-Saharan Africa Populations

**DOI:** 10.1371/journal.pone.0067946

**Published:** 2013-07-05

**Authors:** Eliford Ngaimisi, Abiy Habtewold, Omary Minzi, Eyasu Makonnen, Sabina Mugusi, Wondwossen Amogne, Getnet Yimer, Klaus-Dieter Riedel, Mohammed Janabi, Getachew Aderaye, Ferdinand Mugusi, Leif Bertilsson, Eleni Aklillu, Juergen Burhenne

**Affiliations:** 1 Division of Clinical Pharmacology, Department of Laboratory Medicine, Karolinska Institutet, Stockholm, Sweden; 2 Unit of Pharmacology, School of Pharmacy, Muhimbili University of Health and Allied Sciences, Dar es Salaam, Tanzania; 3 Department of Pharmacology, School of Medicine, Addis Ababa University, Addis Ababa, Ethiopia; 4 Department of Clinical Pharmacology and Pharmacoepidemiology, University of Heidelberg, Heidelberg, Germany; 5 Venhälsan, Karolinska Institutet, Södersjukhuset, Stockholm, Sweden; 6 Department of Internal Medicine, Muhimbili National Hospital, Dar es Salaam Tanzania; 7 Department of Medicine, Unit of Infectious Diseases, Karolinska Institutet, Karolinska University Hospital, Huddinge, Sweden; 8 Department of Internal Medicine, School of Medicine, Addis Ababa University, Addis Ababa, Ethiopia; 9 Department of Internal Medicine, Muhimbili University of Health and Allied Sciences, Dar es Salaam, Tanzania; University of New South Wales, Australia

## Abstract

**Objectives:**

We evaluated the importance of ethnicity and pharmacogenetic variations in determining efavirenz pharmacokinetics, auto-induction and immunological outcomes in two African populations.

**Methods:**

ART naïve HIV patients from Ethiopia (n = 285) and Tanzania (n = 209) were prospectively enrolled in parallel to start efavirenz based HAART. CD4+ cell counts were determined at baseline, 12, 24 and 48 weeks. Plasma and intracellular efavirenz and 8-hydroxyefvairenz concentrations were determined at week 4 and 16. Genotyping for common functional *CYP2B6*, *CYP3A5*, *ABCB1*, *UGT2B7* and *SLCO1B1* variant alleles were done.

**Result:**

Patient country, CYP2B6*6 and *ABCB1 c.4036A>G (rs3842A>G)* genotype were significant predictors of plasma and intracellular efavirenz concentration. CYP2B6*6 and *ABCB1 c.4036A>G* (rs3842) genotype were significantly associated with higher plasma efavirenz concentration and their allele frequencies were significantly higher in Tanzanians than Ethiopians. Tanzanians displayed significantly higher efavirenz plasma concentration at week 4 (p<0.0002) and week 16 (p = 0.006) compared to Ethiopians. Efavirenz plasma concentrations remained significantly higher in Tanzanians even after controlling for the effect of CYP2B6*6 and *ABCB1 c.4036A>G* genotype. Within country analyses indicated a significant decrease in the mean plasma efavirenz concentration by week 16 compared to week 4 in Tanzanians (p = 0.006), whereas no significant differences in plasma concentration over time was observed in Ethiopians (p = 0.84). Intracellular efavirenz concentration and patient country were significant predictors of CD4 gain during HAART.

**Conclusion:**

We report substantial differences in efavirenz pharmacokinetics, extent of auto-induction and immunologic recovery between Ethiopian and Tanzanian HIV patients, partly but not solely, due to pharmacogenetic variations. The observed inter-ethnic variations in efavirenz plasma exposure may possibly result in varying clinical treatment outcome or adverse event profiles between populations.

## Introduction

Sub-Saharan Africa has the highest disease burden of HIV/AIDS worldwide and antiretroviral therapy is widely practiced in the continent. Africa is considered to be the origin of modern human. Sub-Saharan African populations over a longer period of time have acquired vast genetic diversity than any other race in the world, and genetic diversity is reduced with the distance from East Africa [1], [2]. Ethnicity is often associated with varying frequency distribution of variant alleles between populations, which may result in variability in plasma exposure. Moreover there is great environmental and cultural diversity within Africa. For instance Ethiopians are of sematic origin while Tanzanians comprise of Bantu and Nilotic [2]. Although both countries are located in East Africa there is wide environmental and cultural diversity between the two populations. Consequently, the existing wide host genetic and environmental diversity may result in different efficacy and adverse event profiles or treatment outcome between different African populations treated with same ART regimen.

Efavirenz containing combined antiretroviral therapy is the first line treatment for HIV/AIDS in Africa. Efavirenz is primarily metabolized to 8-hydroxyefavirenz mainly by CYP2B6 and to a lesser extent by CYP3A [3]. UGT2B7 is involved in direct N-glucuronidation of efavirenz and O-glucuronidation of 8-hydroxyefavirenz. [4], [5] While *CYP2B6* genotype is the most important genetic factor influencing plasma efavirenz concentration, the importance *UGT2B7* and *CYP3A5* genotype particularly in CYP2B6 slow metabolizers is reported recently [6]–[8]. In vitro and animal studies report that P-glycoprotein and OATP1B1 are not the main cellular transporter proteins for efavirenz. However significant association of *ABCB1 c.4036A>G* with higher plasma efavirenz concentrations in Ugandan healthy volunteers is described [8], a finding latter confirmed in HIV patients from South Africa [9], Uganda [10] and other populations [11]. Accordingly P-glycoprotein may play a role in efavirenz cellular transport in human or alternatively *ABCB1 c.4036A>G* might be in strong linkage disequilibrium with other SNPs located in another gene relevant for efavirenz disposition and hence may serve as tag SNP. OATP1B1, coded by *SLCO1B1*, mediate hepatic uptake of numerous drugs including antiretroviral drugs. *SLCO1B1* genetic polymorphism on efavirenz pharmacokinetics and treatment response among HIV patients remains to be explored. These enzymes and drug transporter proteins are inducible by efavirenz via the activation of human Constitutive Androstane nuclear receptor and human Pregnane X Receptor [12], [13]. All these enzymes, transporter proteins and nuclear receptors involved in efavirenz disposition and inductions are genetically polymorphic, presenting wide between population differences in functional variant allele frequency distribution and protein activity. Genetic variation in drug metabolizing enzymes influencing plasma exposure of the inducer may result in variability in enzyme induction between individuals and populations. Recently we reported that the extent of efavirenz auto induction differ with *CYP2B6*, *UGT2B7* and *CYP3A5* genotype [6], [7], [14]. Although effect of ethnicity in efavirenz pharmacokinetics is well understood, its importance in efavirenz auto-induction remains unclear and needs to be investigated.

It is well recognized that plasma efavirenz pharmacokinetics displays wide inter-individual and inter-ethnic variability [15], [16]. Differences in efavirenz pharmacokinetics may reflect differences in treatment outcomes between populations. Albeit having higher efavirenz plasma concentration, lower virologic response rates in blacks compared to Asian and White populations are reported [15]–[18]. Possibly the associated higher adverse events profile such as liver enzyme abnormality and neuropsychiatric manifestations [19]–[22] may attribute to adherence problem and hence lower treatment outcome in Blacks. Characterization of efavirenz pharmacogenetics, pharmacokinetics, induction and treatment outcomes between different populations would form a base for population specific rationalized efavirenz dose adjustment strategies.

Despite the existence of wide genetic heterogeneity, higher prevalence of HIV and use of ART in Sub-Saharan Africa, the importance of ethnicity, environmental and cultural diversity on efavirenz pharmacokinetics and immunological recovery within Africans is not adequately addressed. In the present study using the same study design, we performed parallel comparative multicenter prospective clinical study to assess the importance of ethnicity, geographic differences and pharmacogenetic variations on efavirenz pharmacokinetic and immunological outcome in HIV patients from two genetically different African populations, Ethiopians and Tanzanians. We report significant differences in efavirenz pharmacogenetic, pharmacokinetics, extent of efavirenz induction and immunologic outcome between the two black African populations.

## Methods

### Ethics Statement

The study protocol was ethically approved by the Institutional Review Board (IRB) of the Muhimbili University of Health and Allied Sciences in Dar es Salaam, Tanzania, by the IRB of Faculty of Medicine, Addis Ababa University and Ethiopian National Ethics Review Committee and by the IRB of Karolinska Institutet in Stockholm, Sweden. Prior written informed consent was obtained from all study participants.

### Study Design and Population

The present study is a part of multi-centered clinical trial project entitled “Optimization of TB-HIV treatment in Africa” financed by European and Developing Countries Clinical Trial Partnership (EDCTP). The study was conducted between September 2007 and June 2010 and registered at Pan African Clinical Trials Registry (registration number: PACTR2009040001261177). A cohort of adult HIV infected patients (n = 494), naïve for ART with CD4 count less than 200 cells/mL were recruited prospectively and enrolled in Addis Ababa, Ethiopia (n = 285) and Dar es-Salaam, Tanzania (n = 209) in parallel. The study participants were local Ethiopian and Tanzanian origin who were residents of Addis Ababa and Dar es Salaam respectively.

### Treatment and Laboratory Analysis

**Figure 1:**
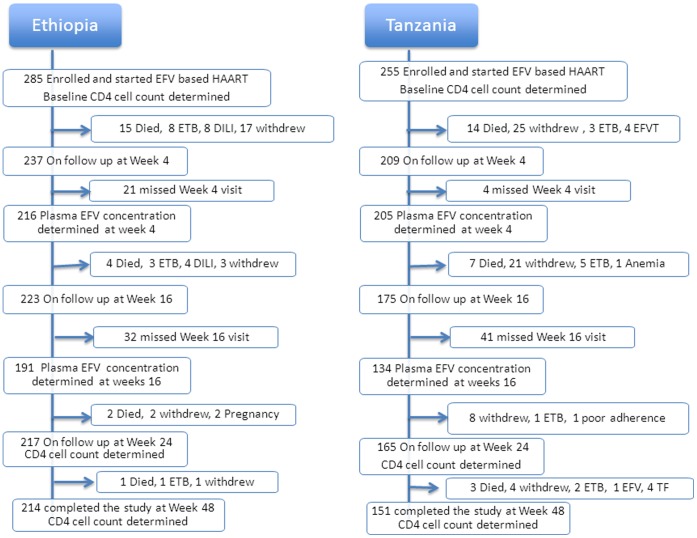
Parallel flow chart of patient enrollment and follow up in Ethiopia and Tanzania during the study period. Acronyms: Withdraw = defaulter or Lost to follow up; EFVT = exited from the study due to efavirenz toxicity; ETB = exited from the study due to tuberculosis (TB) diagnosis; DILI = drug induced liver injury; LTFU = lost to follow-up; Anemia = exited due to anemia and regimen change to nevirapine based HAART; TF = exited after regimen change to second line ARVs due to treatment failure.

After recording baseline clinical, demographic and laboratory parameters, efavirenz-based HAART was initiated with a subsequent clinical and laboratory monitoring for one year. The antiretroviral treatment consisted of stavudine/lamivudine/efavirenz (D4T/3TC/EFV) or zidovudine/lamivudine/efavirenz (AZT/3TC/EFV) or tenofovir/lamivudine/efavirenz (TDF/3TC/EFV). All study participants in both countries were informed to take efavirenz without food at bedtime. Adherence was assessed by self-report. Baseline and follow-up monitoring of clinical and laboratory parameters including plasma/intracellular efavirenz concentrations determinations were conducted in parallel at identical study time points for both study populations (Figure 1). A complete history and physical examination were recorded from each participant before enrolment. Laboratory tests performed before HAART initiation included complete and differential blood counts, platelet count, CD4 count, HIV RNA determination, hepatitis B surface antigen, anti-hepatitis C antibody, serum albumin, renal function tests, liver function tests including; aspartate aminotransferase (AST), alanine aminotransferase (ALT), alkaline phosphatase (ALP), and direct and total bilirubin. The change in CD4 cell counts from baseline during HAART was monitored on 12, 24 and 48 weeks after starting HAART.

### Genotyping for CYP2B6, CYP3A5, UGT2B7, SLCO1B1 and ABCB1

Genomic DNA was isolated from peripheral blood leukocytes using QIAamp DNA Maxi Kit (QIAGEN GmbH. Hilden. Germany). Genotyping for the common functional variant alleles in five relevant genes for efavirenz disposition were carried out at the division of clinical pharmacology, Department of laboratory medicine, Karolinska Institutet Stockholm, Sweden. Genotyping were done by real time PCR using pre-developed Taqman assay reagents for allelic discrimination (Applied Biosystems Genotyping Assays) according to the manufacturer’s instructions. Allelic discrimination reactions were performed using TaqMan® (Applied Biosystems, CA, USA) genotyping assays with the following ID number for each SNP: (C__7586657_20 for *ABCB1* c.3435C>T rs1045642, C__11711730_20 for CYP2B6*6 c.516G>T rs3745274, C__30720663_20 for *UGT2B7* g.-*372G>A* rs7662029 (UGT2B7*2b,*2c,*2d,*2f), C__26201809_30 for CYP3A5*3 c.6986A>G rs776746, C__30203950_10 for CYP3A5*6 14690G>A g.14690G>A, C__32287188_10 for CYP3A5*7 g.27131_27132insT rs241303343, C___1901697_20 for *SLCO1B1* c.388A>G rs2306283 (*1b) and C__30633906_10 for *SLCO1B1* c.521T>C rs4149056 (*5) on ABI 7500 FAST (Applied Biosystems, Foster City, CA). The final volume for each reaction was 10 µl, consisting of 2× TaqMan Universal PCR Master Mix (Applied Biosystems), 20 X drug metabolizing genotype assay mix and 10 ng genomic DNA. The PCR profile consisted of an initial step at 50°C for 2 min and 50 cycles with 95°C for 10 min and 92°C for 15 sec. Genotyping for SLCO1B1 c.388A>G (rs2306283) and c.521T>C (rs4149056) in Tanzanian subjects was done using LightCycler® based method [23]. Haplotype analysis was done using Haploview v.4.1 software. Characterized SNPs were selected on the basis of their potential or identified influence on the functionality of enzymes and transporters proteins obtained from public databases.

### Quantification of Plasma and Intracellular Efavirenz and 8-hydroxy-efavirenz Concentrations

Efavirenz AUC_0–24_ is accurately estimated from single plasma sample obtained at 12 or 16 hours post efavirenz dosing [24]. Four and sixteen weeks after initiation of efavirenz based HAART, 16 h post-dose duplicate blood samples were collected in vacutainer CPT tubes (Becton Dickinson, Heidelberg, Germany). Time of efavirenz intake was inquired before blood withdrawal to make sure 16 h post efavirenz dose blood sample collection. Blood samples were centrifuged (1700 g for 20 min) and plasma and peripheral blood mononuclear cells (PBMC) were prepared as described by Burhenne *et al* [25] and stored at −80°C. Samples were sent on dry ice to the Department of Clinical Pharmacology and Pharmacoepidemiology, University of Heidelberg, Germany where plasma and intracellular efavirenz and 8-hydroxyefavirenz concentrations were determined by liquid chromatography-tandem mass spectrometry (LC/MS/MS) as described previously [6], [25]. Efavirenz and 8-hydroxyefavirenz were quantified using ^13^C_6_-efavirenz and ^2^H_4_-8-hydroxyefavirenz as internal standards and electrospray tandem mass spectrometry. The lower limits of quantification in plasma were 10.0 ng/mL for efavirenz and 0.4 ng/mL for 8-hydroxyefavirenz. The efavirenz (8-hydroxyefavirenz) calibration range was 10–10000 ng/mL (0.4–400 ng/mL). Linear regression with 1/x weighing resulted in correlation coefficients of r^2^>0.99. Accuracy and precision (within-batch and batch-to-batch) of the assay fulfilled all recommendations of FDA guidelines.

### Statistical Analyses

For baseline characteristics, independent group t-test and chi-square test were used for comparison of continuous and categorical variables, respectively. Efavirenz plasma concentration data were log 10 transformed to achieve normality of data distribution. Mean plasma efavirenz concentration between and within countries over time was assessed using independent t-test and paired sample t-test respectively. Factors influencing Efavirenz plasma and intracellular levels were assessed by hierarchical multivariate linear regression model building in SPSS. First, Univariate linear regression analyses were used to identify variables that significantly influenced efavirenz plasma levels. Variables at p-value less than 0.1 were hierarchically entered into the multivariate analysis and a p value <0.05 were considered a significant predictor in the final model. Factors influencing CD4 gain by week 12, 24 and 48, respectively were assessed by hierarchical multivariate linear regression model building in SPSS. A change in CD4 count from baseline was used as a dependent variable and linear regression model building procedure was implemented as stated above. Nonlinear mixed effect modeling for absolute CD4 count- time profile was performed using NonMem version 7.2 and step wise covariate model building was implemented using PsN 3.5.3. Repeated measures ANOVA was performed to compare mean CD4 counts between treatment weeks and to determine any interaction between patient country, *CYP2B*6 genotypes and duration of therapy.

## Results

### Patient Characteristics

The demographic and baseline clinical characteristics of study participants stratified by study population are presented in Table 1. Flow chart of patient enrollment and follow up through the study period in Ethiopia and Tanzania is presented in Figure 1.

**Table 1:** Baseline demographic, clinical and laboratory characteristics of study patients.

Parameters	Ethiopia		Tanzania	
	N	Value	N	Value
Proportion of female (%)	285	72.6	209	66.6
Proportion with HBsAg (%)	285	5.6		1.4
Proportion with HCV (%)	285	2.4		3.8
Types of HAART prescribed	285		104	
AZT/3TC/EFV (n,%)		117 (41.1)		94 (90.4)
d4T/3TC/EFV (n,%)		156 (54.7)		10 (9.6)
TDF/3TC/EFV (n,%)		3 (4.2)		0 (0)
Median age in years	285	34	209	37
Median weight Kg	285	51	208	56
Median BMI	285	19.3	205	22
Median hemoglobin	272	12.6	195	10.6
Median Blood platelet count Cells/µL	268	233	190	263
Median AST U/L	281	33	189	32
Median ALT U/L	281	28	199	23
Median ALP U/L	280	108.5	161	83
Median total bilirubin µmol/L	281	8.0	174	6.7
Median plasma albumin g/dL	246	40	197	41
Median Serum creatinine µmol/L	243	70.7	202	78
Median baseline CD4 Cells/µL	285	103	209	95
Median baseline HIV RNA/mL	232	159000	164	182699

HBsAg = hepatitis B surface antigen, HCV = hepatitis B antibody, HAART = highly active antiretroviral combination therapy, AZT = zidovudine, 3TC = lamivudine, EFV = efavirenz, d4T = stavudine, TDF = tenofovir, BMI = body mass index, Hb = blood hemoglobin, AST = aspartate aminotransferase, ALT = alanine aminotransferase, ALP = alanine phosphotransferase.

### CYP2B6, CYP3A5, UGT2B7, ABCB1 and SLCO1B1genotype

Genotype frequencies were determined in Ethiopian (n = 262) and Tanzanian patients (n = 184). Haplotype analyses indicated no linkage between *CYP3A5* single-nucleotide polymorphisms. Hence for the statistical analysis, subjects were grouped on the basis of the number of functional *CYP3A5* alleles (CYP3A5 *1). Comparison of genotype and allele frequencies distribution between the two populations for the common functional variant alleles in *CYP2B6*, *CYP3A5*, *UGT2B7*, *ABCB1* and *SLCO1B*1 genes is presented in Table 2. The frequencies of all variant alleles determined in this study were significantly different between the two populations.

**Table 2:** Comparison of genotype and variant allele frequency distribution between Ethiopian and Tanzanian HIV patients.

Genotype		Ethiopia	Tanzania	?2	P value
		N (%)	N (%)		
CYP2B6 c.516G>T(*6)	GG	121 (45.8%	64 (35.0%)		
	GT	120 (45.5%)	85 (46.4%)	11.4	0.003
	TT	23 (8.7%)	34 (18.6%)		
Number of CYP3A5*1 allele	Zero	157 (59.5%)	48 (26.2%)		
	One	92 (34.8%)	93 (50.8%)	58.0	<0.0001
	Two	15 (5.7%)	42 (23.0%)		
*UGT2B7 -372G>A*	AA	55 (21.0%)	16 (8.7%)		
	AG	143 (54.6%)	76 (41.3%)	34.4	<0.0001
	GG	64 (24.4%)	92 (50.0%)		
*ABCB1 c.3435*	TT	13 (4.9%)	2 (1.1%)		
	CT	90 (34.1%)	53 (29.0%)	6.96	0.03
	CC	161 (61.0%)	128 (69.9%)		
*ABCB1 c.4036AG (rs3842)*	AA	193 (73.7%)	108 (58.7%)		
	AG	62 (23.7%)	71 (38.6%)	11.7	0.003
	GG	7 (2.7%)	5 (2.7%)		
SLCO1B1 D130N (*1b)	AA	38 (14.5%)	4 (2.2%)		
	AG	127 (48.5%)	41 (22.5%)	66.1	<0.0001
	GG	97 (37.0%)	137 (75.3%)		
SLCO1B1 A174V (*5)	CC	18 (6.9%)	0		
	CT	74 (28.2%)	11 (6.1%)	51.6	<0.0001
	TT	170 (64.9%)	170 (93.9%)		
**Allele**	**Minor allele**	**Allele frequencies (%)**			
CYP2B6 c.516G>T(*6)	*T*	31.4	41.8	10.1	0.001
CYP3A5 *3	*3	64.0	20.5	165	<0.0001
CYP3A5 *6	*6	12.9	20.8	9.95	0,002
CYP3A5 *7	*7	0	10.4	–	–
*ABCB1 c.3435C>T*	*T*	22.0	15.5	5.67	0,011
*ABCB1 c.4036AG (rs3842)*	*G*	14.5	22.0	11.7	0,004
*UGT2B7 -327 G>A*	*A*	48.3	29.3	32.2	<0.0001
SLCO1B1 D130N (*1b)	*1b	38.7	13.5	67.5	<0.0001
SLCO1B1 A174V (*5)	*5	21.0	0.0	58.5	<0.0001

### Factors Influencing Plasma Efavirenz Concentrations

**Figure 2:**
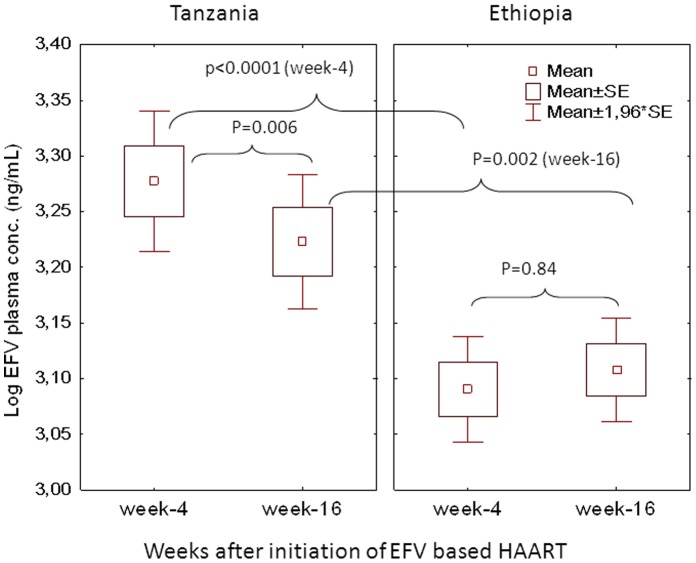
Comparison of mean ± SE of mean efavirenz plasma concentration on the 4^th^ and 16^th^ weeks after initiation of efavirenz based HAART separately between Ethiopian and Tanzanian patients using independent t test. Comparison of efavirenz concentration between week 4 and week 16 with in Ethiopians and Tanzanian patients was done using paired t test. Boxes indicate mean ± SE of the mean; bars indicate mean ±1.96 × SE of the mean.

**Figure 3:**
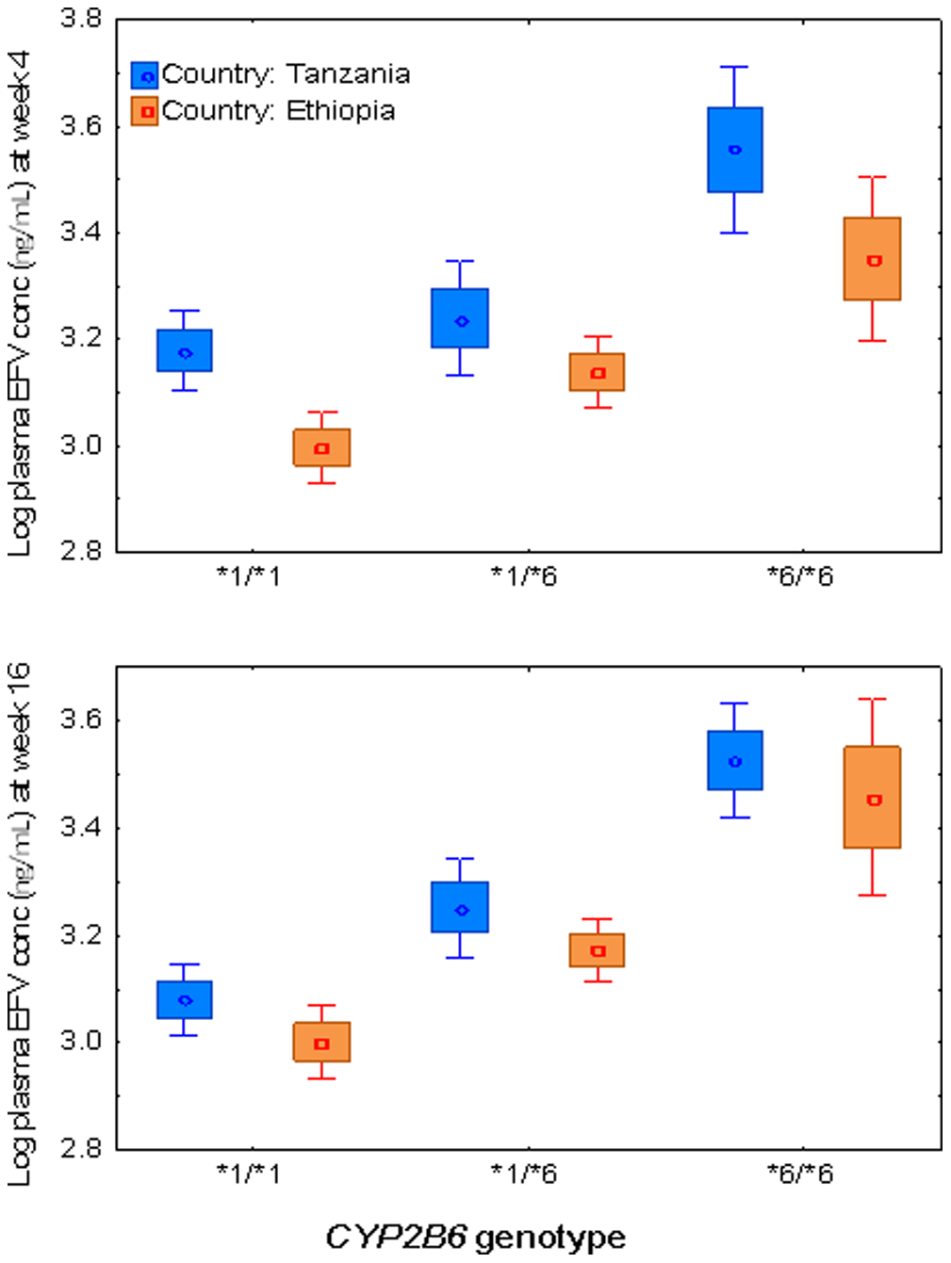
Comparison of mean log efavirenz plasma concentrations at week 4 and week 16 of efavirenz therapy between Ethiopian and Tanzanian HIV patients having the same CYP2B6*6 genotype. Boxes indicate mean ± SE of the mean; bars indicate mean ±1.96 × SE of the mean.

Comparison of mean ± SE of log plasma efavirenz concentration at week 4 and 16 stratified by country is presented in Figure 2. Independent t test indicated higher mean log plasma efavirenz concentration in Tanzanians than Ethiopians at both week 4 (p<0.0001) and 16 (p = 0.002). Plasma efavirenz concentration at both study time points were significantly higher in Tanzanians compared to Ethiopians even after controlling for effect of *CYP2B6* genotype (Figure 3). Comparison of median efavirenz plasma concentration at week 4 and week 16 separately between Ethiopian and Tanzanian HIV patients in all and stratified by genotype is presented in Table 3. Within country analyses using paired samples t test indicated a significant decrease in the mean plasma efavirenz concentration at week 16 than week 4 in Tanzanians (p = 0.006), whereas no significant differences in plasma concentration over time was observed in Ethiopians (p = 0.84).

**Table 3:** Comparison of median plasma efavirenz concentrations between Ethiopian and Tanzanian HIV patients (in all and stratified by genotypes) at week-4 and week-16 after initiation of efavirenz based HAART.

		Plasma efavirenz concentration at week-4 (µg/L)					Plasma efavirenz concentration at week-16 (µg/L)				
		Ethiopia		Tanzania			Ethiopia		Tanzania		
		n	Median (IQR)	n	Median (IQR)	p	n	Median (IQR)	n	Median (IQR)	p
All		215	1208 (831–1857)	205	1892 (1235–4026)	<0.0001	191	1276 (884–1860)	134	1584 (1112–2600)	0.003
CYP2B6*6											
	*1/*1	94	1018 (767–1500)	63	1472 (1021–2117)	0.001	89	1124 (773–1528)	43	1216 (895–1688)	0.11
	*1/*6	97	1338 (1052–2025)	84	1814 (1326–3113)	0.045	87	1425 (985–1963)	57	1588 (1270–2600)	0.16
	*6/*6	16	2670 (1177–4055)	33	4595 (3347–5815)	0.07	11	3307 (1111–4259)	21	3381 (2566–4374)	0.53
UGT2B7*2											
	AA	43	1159 (826–1880)	15	3118 (2096–4984)	0.004	36	1136 (809–1955)	9	2566 (1151–4294)	0.03
	AG	114	1211 (860–1837)	75	1861 (1182–4026)	0.04	105	1369 (985–1881)	51	1610 (1125–2600)	0.06
	GG	50	1234 (767–1841)	91	1780 (1279–3735)	0.002	46	1264 (831–1633)	63	1491 (1081–2201)	0.02
Number of CYP3A5*1 allele											
	0	124	1192 (829–1852)	47	1657 (943–4234)	0.46	111	1245 (934–1805)	32	1841 (1217–4444)	0.20
	1	73	1208 (824–1764)	91	1974 (1173–4501)	0.000	68	1285 (819–1854)	65	1649 (1156–2566)	0.06
	2	10	1380 (1038–3377)	42	1730 (1363–2699)	0.32	8	2191 (1566–3848)	24	1447 (1095–1967)	0.003
ABCB1 c.3435 C/T											
	TT	11	1109 (755–1880)	2	1381 (281–2480)	0.000	8	1248 (784–1936)	2	938 (175–1700)	0.001
	CT	70	1179 (823–2060)	53	1887 (1235–4232)	0.04	58	1397 (1029–1965)	34	1605 (1112–3381)	0.12
	CC	126	1215 (902–1837)	125	1892 (1279–3801)	0.99	121	1235 (832–1839)	85	1580 (1156–2566)	0.60
ABCB1 rs3842 A/G											
	AA	150	1178 (826–1787)	107	1767 (1182–4203)	0.0003	137	1233 (869–1839)	71	1649 (1156–3345)	0.00
	AG	52	1468 (900–2127)	69	2041 (1413–4171)	0.004	46	1525 (984–1947)	50	1518 (1081–2409)	0.22
	GG	5	1183 (1109–1547)	5	1933 (39–2466)	0.48	4	1406 (1072–1939)	2	1404 (1151–1657)	0.94
SLCO1B1 D130N (*1b)											
	AA	28	1243 (836–1612)	4	3172 (1351–5440)	0.14	28	1439 (882–1714)	3	1439 (1096–4403)	0.41
	AG	99	1174 (860–1837)	39	2440 (1530–4595)	0.007	89	1270 (880–1839)	27	1700 (1282–2634)	0.11
	GG	80	1298 (821–2184)	136	1802 (1175–3955)	0.01	70	1323 (973–2024)	91	1491 (1112–2600)	0.03
SLCO1B1 A174V (*5)											
	CC	13	1074 (755–2515)	0			15	934 (636–1389)	0		
	CT	59	1233 (842–2060)	10	1424 (741–3063)	0.45	52	1428 (967–2572)	9	1477 (1184–1995)	0.38
	TT	135	1208 (860–1837)	168	1892 (1292–4218)	<0.0001	120	1292 (940–1735)	112	1584 (1127–2718)	<0.0001

Log transformed efavirenz plasma concentration was used in ANOVA test.

Efavirenz plasma levels at week 4 and 16 from both Ethiopian and Tanzanian HIV patients were pooled to make a single dependent variable in order to evaluate any interaction between duration of therapy and patient country in influencing efavirenz steady state concentration. Time on therapy (week at which efavirenz plasma levels assessed) was not a significant predictor (p = 0.35). However, when time on efavirenz therapy was tested for interaction with other possible variables affecting efavirenz plasma level; significant interaction with country was found (p = 0.02). Therefore further assessments of factors affecting efavirenz plasma level at week 4 and week 16 were done separately (not using the pooled data). Univariate linear regression analysis identified the following variables as predictors of efavirenz plasma level at week 4; CYP2B6*6 genotype (9.8%, p<0.0001), country (4.8%, p<0.0001), co infection with Hepatitis B at recruitment (0.6%, p = 0.061), CYP3A5*1 genotype (1.5%, p = 0.019), *ABCB1 c.3435C>T* (0.8%, p = 0.082), *ABCB1* c.4036A>G (2.4%, p = 0.003), SLCO1B1*1b (0.8%, p = 0.084). Results of hierarchical multivariate regression analysis for efavirenz plasma level at week 4 are presented in Table 4. Only *CYP2B6* genotypes (p<0.0001), country (p = 0.035), and *ABCB1* c.4036A>G genotypes (p = 0.002) were associated with significant increase in model explained inter-individual variability of week 4 efavirenz plasma level. The overall model explained for 16% of inter-individual variability in week 4 efavirenz plasma levels (p<0.0001). When hierarchical models were built for each country separately, CYP2B6*6 contributed 8.3% and 11% while ABCB1 c.4036A>G contributed 0.6% and 8.3% of the inter-individual variability in week 4 plasma efavirenz concentration in Ethiopian and Tanzanian patients respectively.

**Table 4:** Multiple linear regression models for various study dependent variables.

Model	Dependent variable	Explicative variables	Beta	Partial R^2^	R^2^	p value
1	Log EFV at wk4	(Constant)	3.075		0.16	<0.001
		*ABCB1c.3842CT*	0.437	0.01		0.052
		*ABCB1c.3842CC*	0.525	0.02		0.004
		CYP2B6*1/*6	0.16	0.03		0.002
		CYP2B6*6/*6	0.324	0.1		<0.001
		Country	0.12	0.01		0.035
2	Log EFV at wk16	(Constant)	2.994		0.202	<0.001
		HBsAg+ve	0.129	0.02		0.014
		CYP2B6*1/*6	0.26	0.07		<0.001
		CYP2B6*6/*6	0.401	0.15		<0.001
		Country	0.091	0.01		0.084
5	Log IC EFV at wk4	(Constant)	1.37		0.38	<0.0001
		Log [EFV] wk4	0.529	0.27		<0.0001
		Country	0.143	0.02		0.041
6	Log IC EFV at wk16	(Constant)	1.616		0.324	<0.0001
		Log [EFV] wk16	0.434	0.18		<0.0001
		HBsAg+ve	0.099	0.01		0.094
		Country	0.172	0.04		0.006
7	CD4 gain at wk12	(Constant)	−29		0.051	
		Log [IC EFV] wk4	0.196	0.038		0.004
		Country	0.134	0.016		0.059
8	CD4 gain at wk24	(Constant)	83		0.07	
		CD4 count [wk0]	−0.225	0.054		<0.0001
9	CD4 gain at wk48	(Constant)	94		0.14	
		CD4 count [wk0]	−0.205	0.036		0.008
		Country	0.259	0.056		0.002

IC EFV = intracellular efavirenz, EFV = efavirenz.

Univariate linear regression analysis identified the following variables as predictors (*r* ^2^, p value) of efavirenz plasma level at week 16; CYP2B6*6 genotype (17.9%, p<0.0001), country (2.5%, p<0.0001), coinfection with Hepatitis B at recruitment (2.6%, p = 0.003). As shown in Table 4, only CYP2B6*6 genotypes (p<0.0001) and baseline hepatitis B co infection (p = 0.002) were associated with significant increase in model explained inter-individual variability of week 16 efavirenz plasma concentration. Country had a trend to influence variability in efavirenz plasma level at week 16 (p = 0.08). The model explained for 20% of inter-individual variability in week 16 plasma efavirenz levels (p<0.0001). On separate model building for each country, CYP2B6*6 contributed 13.3% and 20.6% of inter-individual variability in efavirenz plasma level at week 16 in Ethiopian and Tanzanian patients, respectively.

### Factors Determining Efavirenz Intracellular Concentrations

Factors influencing efavirenz intracellular levels individually at week 4 were; efavirenz plasma concentrations (29.2%, p<0.0001), country (11.9%, p<0.0001), CYP2B6*6 genotypes (4%, p = 0.02), body mass index (2.2%, p = 0.009), baseline ALT levels (1.9%, p = 0.014), SLCO1B1*5 (1.8%, p = 0.039), SLCO1B1*1b (1.5%, p = 0.056) and CYP3A5*1 (3.6%, p = 0.004). In multivariate model, only efavirenz plasma concentrations at week 4 (p<0.0001) and country (p = 0.041) remained significant predictor of intracellular efavirenz levels at week 4. Independent variable coefficients in the final model are indicated in Table 4. Despite dependence of plasma efavirenz levels on CYP2B6*6 genotype, it remained a significant model predictor for intracellular plasma level in each genotype after stratified multiple regression analysis. The model explained for 38% of inter-individual variability in week 4 intracellular efavirenz levels (p<0.0001).

Factors influencing efavirenz intracellular levels individually at week 16 were; efavirenz plasma levels at week 16 (25%, p<0.0001), country (10.2%, p<0.0001), CYP2B6*6 (6.9%, p<0.0001), SLCO1B1*1b (2.1%, p = 0.034), SLCO1B1*5 (1.5%, p = 0.056) baseline Hepatitis B co infection (2.1% p = 0.014). However, only efavirenz plasma levels at week 16 and country were significant predictors of the intracellular efavirenz levels. The model explained for 32.4% of inter-individual variability in week 16 intracellular efavirenz levels (p<0.0001).

### Immunological Outcomes between Ethiopians and Tanzanians

**Figure 4:**
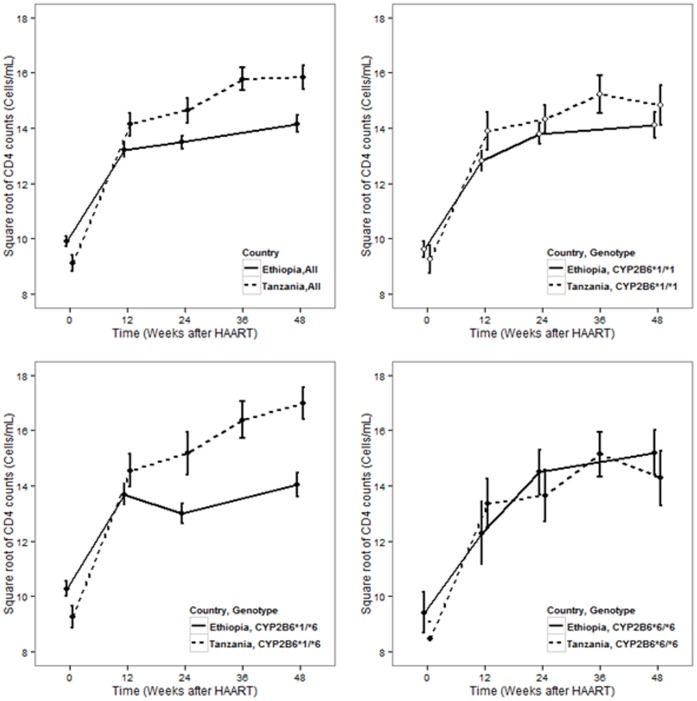
Standard error bar plots for CD4 gain profile among Ethiopian compared to Tanzanian patients, all and stratified by *CYP2B6* genotype.

**Figure 5:**
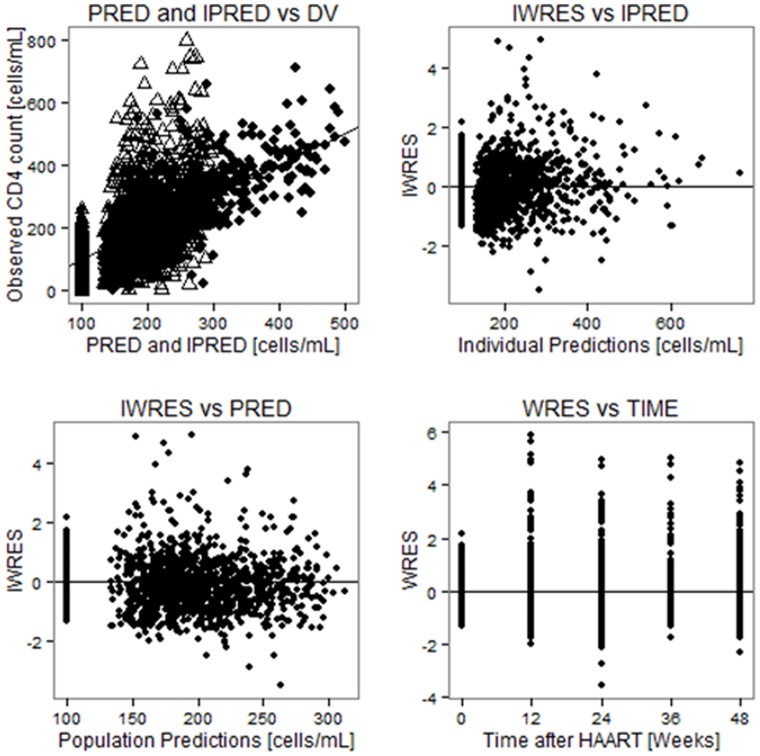
Basic goodness of fit plots for the final model, PRED and IPRED versus DV are plotted on same graph with IPRED VS DV shown by black, square points. PRED = Population prediction, IPRED = Individual prediction, DV = Observed CD4 cell counts, WRES = Weighted residuals, IWRES = Individual weighted residuals.

Both by week 24 (p = 0.004) and week 48 (p = 0.002) of efavirenz therapy the mean CD4 cell count was significantly higher in Tanzanians than Ethiopians (Figure 4). Within subject tests of repeated measure ANOVA showed a significant increase in CD4 with time (p<0.0001). Bonferroni post hoc test indicated that major increases were between week 0 and 12 (p<0.0001) and between week 24 and 48 (p = 0.008). There was no significant difference between week 12 and 24 (p = 0.57). Between subject effect tests of repeated measure ANOVA showed a significant effect for country (p = 0.004) but not for *CYP2B6* (p = 0.29). Splitting the data by country, between subject effect test for *CYP2B6* genotype was not significant for Tanzanians (p = 0.52). There was, however, a trend of having higher CD4 gain for being carrier of CYP2B6*6 allele in gene dose dependent manner for Ethiopians (p = 0.11). Having the same *CYP2B6* genotype, Tanzanians displayed higher CD4 gain than Ethiopians except in CYP2B6*6 genotype groups (see Figure 4). As shown in Table 4, hierarchical multivariate linear regression model building indicated intracellular efavirenz concentrations at week 4 (p = 0.013) to be a significant predictor of CD4 gain by week 12, while patient country (p = 0.059) had a trend. Stepwise covariate model building implemented using Pearl speaks NonMem (PsN) to identify factors influencing model parameter values was used as a covariate model building strategy. As shown in Table 5, maximum gain in CD4 count (Emax) was higher for individuals who had baseline absolute CD4 count higher than 98 (a median value); it was also higher for Tanzanian patients compared to Ethiopians. Figure 5 shows the goodness of fit plots for the final model and model parameter values are given in Table 5.

**Table 5:** Parameter values for final nonlinear mixed effect model for absolute CD4 count gain after initiation of HAART among HIV patients (n = 492).

Parameter	Description	Unit	Typical value (RSE)	BSV (RSE)
Base	Estimate of baseline absolute CD4 count	Count/mL	100.4 (2.3)	0‡ (N.E†)
Emax	Estimate of maximum gain in absolute CD4 count	Count/mL	79.7 (5.5)	57 (14)
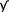	Steepness/sigmoidity of gain in absolute CD4 count	–	0.2‡ (N.E†)	93 (5.3)
T50	Time at which half of maximum CD4 gain is achieved	Days	235 (2.5)	0‡ (N.E†)
ADD	Additive residual error	Count/mL	74.3 (3.0)	–
EmaxCD40	Fractional increase/decrease in Emax for every difference between baseline CD4 count and median value of 98	–	0.0055 (7.3)	–
EmaxSITE	Fractional increase in Emax for Tanzanian subjects	–	0.495 (22.3)	–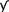 CD40	Fractional increase/decrease in steepness for every baseline CD4 difference from median value of 98	–	−0.0025 (8.5)	–
Model 1	Structural model	OFV	16182	–
Model 2	Structural+covariate model	OFV	16080	–

OFV = objective function value, BSV = between subject variability, N.E† = Not estimated, ‡ = Fixed to this value.

## Discussion

The present study investigated factors determining between patient variability in efavirenz plasma and intracellular concentration as well as immunologic outcome in HIV patients receiving ART from East Africa. Our main finding includes that geographic differences (patient country), CYP2B6*6 and *ABCB1 c.4036A/G* (rs3842) genotypes are significant predictors of efavirenz plasma and intracellular concentrations. We found significant differences in the frequency distributions of all common *CYP2B6*, *CYP3A5*, *UGT2B7*, *SLCO1B1* and *ABCB1* genotypes and variant alleles between Ethiopian and Tanzanian HIV patients (Table 2). The frequency distribution of both CYP2B6*6 and *ABCB1 c.4036A/G,* the two variant alleles associated with higher efavirenz plasma concentrations, was significantly higher in Tanzanians than Ethiopians. Likewise efavirenz plasma and intracellular concentration as well as immunological gain were significantly different between the two populations; being higher in Tanzanians than Ethiopians. The extent of efavirenz auto induction in reducing its plasma concentration over time was pronounced in Tanzanians where as no significant change was observed in Ethiopians (Figure 1). To the best of our knowledge, this is the first study to extensively compare and contrast efavirenz plasma and intercellular pharmacokinetics and change in CD4 gain overtime controlling for the effect of pharmacogenetic variations between two black African populations using the same study design.

Coherent with the observed higher efavirenz plasma concentrations, we noticed more frequent neuropsychiatric symptoms during early initiation of efavirenz treatment among Tanzanian study participants. Five Tanzanian study participants had to discontinue their treatment because of intolerable neuropsychiatric symptoms. In contrast among Ethiopian study participants, we noticed that neuropsychiatric symptoms were less common and none of the patients discontinued their treatment due to CNS related problems. The safety profile and associated risk factors particularly with respect to drug induced liver enzyme abnormality and mortality from the same cohort is published recently [19], [21], [26]. In brief, higher efavirenz plasma concentration and CYP2B6*6 genotype were associated with efavirenz based HAART induced liver enzyme abnormality. Most deaths were associated with advanced HIV disease and the risk factors were oral candidiasis and Kaposi’s sarcoma [26]. No mortality due to suicide was observed.

Study participants were genotyped for common functional variant alleles in five identified or potentially relevant genes for efavirenz disposition. The frequency distributions of all variant alleles were significantly different between the two populations (Table 2). Our finding confirms that at least East Africans display wide pharmacogenetic heterogeneity, and pharmacogenetic data from one geographic region may not be directly extrapolated to others with in the continent [27], [28].

Ethnicity, CYP2B6*6 and *ABCB1 c.4036A/G* were significant predictors of efavirenz pharmacokinetics accounting together for about 20% of between patient variability in efavirenz plasma concentration. Apparently Ethiopians displayed lower efavirenz plasma concentration and hence higher CYP2B6 activity than Tanzanians. This is partly due to higher frequency of variant alleles associated with higher efavirenz plasma concentrations (CYP2B6*6 and *ABCB1 c.4036A/G)* in Tanzanians than Ethiopians. However having the same *CYP2B6* and *ABCB1 c.4036A/G* genotype, Tanzanians had higher median efavirenz plasma and intracellular concentrations as compared to Ethiopians (Table 3). Other unidentified ethnic associated genetic variations and/or environmental factors including dietary habits may account for the observed differences in efavirenz plasma concentration between the two populations. Effect of *CYP2A6* genetic polymorphism was not investigated in the present study and its variant alleles frequencies may differ between the two populations. However contribution of CYP2A6 for efavirenz disposition is minor and *CYP2A6* genetic variation is relevant only in *CYP2B6* slow metabolizers [29] or insignificant [11], [30], [31]. We previously reported higher CYP3A activity in healthy Ethiopians compared to Tanzanians, Swedes and Koreans using 4-betahydroxycholestrol as a CYP3A marker [32]. Our study further reveals the importance of gene-gene and gene-environment interplay determines population variability in the ultimate efavirenz plasma concentrations.

CYP2B6*6, the variant allele associated with high plasma efavirenz concentration, is more frequent in blacks as compared to Hispanics, European and Asian populations [27]. Even within Africans the frequency of CYP2B6*6 varies greatly being much higher in Zimbabweans (49%) than Ugandans (35%) [8], [33]. Accordingly the frequency of CYP2B6*6 allele was significantly higher in Tanzanians (41%) than Ethiopians (31%). The relevance of CYP2B6*6 variant allele in explaining between patient variability in efavirenz pharmacokinetics became pronounced over time in both populations. CYP2B6*6 genotype explained 8.3% and 11% inter-individual variability at week 4 efavirenz plasma level in Ethiopian and Tanzanian patients, respectively. Whereas at week 16, its contribution increased to 13.3% and 20.6% in explaining inter-individual variability in efavirenz plasma level in Ethiopian and Tanzanian patients, respectively.

*ABCB1 c.4036A/G* was also a significant determinant of efavirenz plasma concentration in both Ethiopian and Tanzanian populations. The association of ABCB1 *c.4036A/G* with higher plasma efavirenz concentration was first reported in Ugandan populations [8], a finding latter confirmed in South African [9] and other populations [11]. The present study further confirms the importance of ABCB1 *c.4036AG* genotype in determining plasma efavirenz concentration in Ethiopians and Tanzanian population. Accordingly the variant allele ABCB1 *c.4036AG* should be taken into consideration in addition to *CYP2B6* genotype in pharmacogenetic-based efavirenz dosage optimization in African population.

Influence of *CYP2B6* genotype on the extent of *CYP2B6* induction by efavirenz in Tanzanian and Ethiopian HIV patients were reported previously [6], [7] and recently on CYP3A induction by efavirenz [14]. In the present study data from the two studies were merged with additional more sample size to test the effect of genetic and environmental factors on the extent and duration of efavirenz auto-induction. Our results also show that both country of residence and *CYP2B6* genotypes were the main predictor of the extent of efavirenz auto- induction and hence its influence on efavirenz plasma exposure over time. The decrease in efavirenz concentration over time might considered to be due to lack of adherence. However, more than 95% of the study participants reported that they took their medication regularly. Furthermore we measured not only the concentration of the parent drug efavirenz but also its major primary metabolite, 8-hydroxyefavirenz concentrations. The decrease in efavirenz concentration was accompanied by an increase in 8-hydroxyefavirenz, indicating elevations in efavirenz metabolism. We reported previously a significant correlation between plasma efavirenz concentration and efavirenz metabolic ratio defined as efavirenz/8-hydroxyefvaurenz ratio [6], [7]. Therefore we attribute the decrease in efavirenz plasma concentration to auto induction but not due to lack of adherence. The extent of decline in efavirenz plasma concentrations and metabolic ratios over time due to efavirenz auto induction was more prominent in Tanzanian than Ethiopian patients. Due to this effect the difference in efavirenz plasma concentration at week-16 between the two study populations was not as wide as what was observed at week-4 of efavirenz therapy (Figure 2). Efavirenz induces CYP2B6 mainly via human constitutive androstane receptor (CAR) and pregnane X receptor (PXR) nuclear hormone receptors encoded by NR1I3 and NR1I2 genes, respectively [13]. Differences in foods, spices and beverages consumed in Ethiopia and Tanzania may cause differential activation of hCAR and hPXR and thus varying basal and induced CYP2B6 enzyme expression level. On the other hand both hCAR and hPXR are genetically polymorphic and differences in variant allele frequencies between the two populations may account for the observed variation in the extent of efavirenz auto induction between the two populations. Our study is the first to report variation in the extent of efavirenz auto-induction between two populations.

Individuals with hepatitis B infection at recruitment had higher plasma efavirenz levels both at week 4 and 16 making patient Hepatitis B status a significant predictor of efavirenz plasma concentrations. Efavirenz is mainly metabolized in the liver; therefore impaired liver function may increase efavirenz plasma level. Hence, monitoring of efavirenz plasma concentrations in patients with liver diseases is, therefore, recommended. Recently we reported association of high efavirenz plasma levels and CYP2B6*6 genotype with efavirenz based HAART induced liver enzyme abnormality in HIV patients [19]–[21].

In line with having higher plasma and intracellular efavirenz concentration, the mean CD4 gain by week 24 and 48 was significantly higher in Tanzanian than Ethiopian HIV patients. Due to antiretroviral treatment, HIV viral load decreases and destruction of CD4 cell diminishes at a rate that depends on the degree of arrest on HIV reproduction. Host genetic variability may determine the immune response to HIV and immune reconstitution following initiation of HAART. We reported recently the association of higher β-defensin copy number with increased HIV load prior to HAART and poor immune reconstitution following initiation of HAART [34]. The degree of arrest on HIV reproduction depends on antiretroviral exposure, while the rate of CD4+ production is influenced by genetic, immunological, physiological, and behavioral factors [35]–[38]. It has been reported that in African adults, CD4+ cell counts vary within and among populations [35]. Therefore, the influence of patient country or ethnicity to CD4+ gain in this study can possibly be attributed to both: differences in drug exposure between the two populations and differences in host genetic, environmental or physiological factors influencing the rate of CD4+ cells production.

In summary, we report substantial differences in efavirenz plasma and intra cellular systemic exposure as well as immunologic recovery between Ethiopian and Tanzanian HIV patients receiving similar ART, partly but not solely, due to pharmacogenetic variations between the two populations. Our stud demonstrated not only the existence of between population differences in efavirenz pharmacokinetics and pharmacogenetics but also variations in the extent of efavirenz auto-induction between populations. We emphasize the importance of ethnicity and environment factors in addition *CYP2B6* and *ABCB1* genotype to be considered for efavirenz dosage optimization strategy. Interactions of genetic and environmental factors play a crucial role in determining efavirenz plasma level, which may in turn influence the immunological outcomes. Accordingly the observed ethnic differences in plasma efavirenz exposure may possibly result in between population variability in clinical outcome and treatment safety profile.

## References

[1] Prugnolle F, Manica A, Balloux F (2005) Geography predicts neutral genetic diversity of human populations. Curr Biol 15: R159–160. 15753023 10.1016/j.cub.2005.02.038 PMC1800886

[2] Cavalli-Sforza LL MP, Piazza A (1994) The History and Geography of Human Genes. New Jersy: Princeton University press. 535 p.

[3] Ward BA, Gorski JC, Jones DR, Hall SD, Flockhart DA, et al (2003) The cytochrome P450 2B6 (CYP2B6) is the main catalyst of efavirenz primary and secondary metabolism: implication for HIV/AIDS therapy and utility of efavirenz as a substrate marker of CYP2B6 catalytic activity. J Pharmacol Exp Ther 306: 287–300. 12676886 10.1124/jpet.103.049601

[4] Belanger AS, Caron P, Harvey M, Zimmerman PA, Mehlotra RK, et al (2009) Glucuronidation of the antiretroviral drug efavirenz by UGT2B7 and an in vitro investigation of drug-drug interaction with zidovudine. Drug Metab Dispos 37: 1793–1796. 19487252 10.1124/dmd.109.027706 PMC2729325

[5] Cho DY, Ogburn ET, Jones D, Desta Z (2011) Contribution of N-glucuronidation to efavirenz elimination in vivo in the basal and rifampin-induced metabolism of efavirenz. Antimicrob Agents Chemother 55: 1504–1509. 21282425 10.1128/AAC.00883-10 PMC3067141

[6] Ngaimisi E, Mugusi S, Minzi OM, Sasi P, Riedel KD, et al (2010) Long-term efavirenz autoinduction and its effect on plasma exposure in HIV patients. Clin Pharmacol Ther 88: 676–684. 20881953 10.1038/clpt.2010.172

[7] Habtewold A, Amogne W, Makonnen E, Yimer G, Riedel KD, et al (2011) Long-term effect of efavirenz autoinduction on plasma/peripheral blood mononuclear cell drug exposure and CD4 count is influenced by UGT2B7 and CYP2B6 genotypes among HIV patients. The Journal of antimicrobial chemotherapy 66: 2350–2361. 21846671 10.1093/jac/dkr304

[8] Mukonzo JK, Roshammar D, Waako P, Andersson M, Fukasawa T, et al (2009) A novel polymorphism in ABCB1 gene, CYP2B6*6 and sex predict single-dose efavirenz population pharmacokinetics in Ugandans. Br J Clin Pharmacol 68: 690–699. 19916993 10.1111/j.1365-2125.2009.03516.x PMC2791975

[9] Swart M, Ren Y, Smith P, Dandara C (2012) ABCB1 4036A>G and 1236C>T Polymorphisms Affect Plasma Efavirenz Levels in South African HIV/AIDS Patients. Front Genet 3: 236. 23133441 10.3389/fgene.2012.00236 PMC3488761

[10] Mukonzo JK, Okwera A, Nakasujja N, Luzze H, Sebuwufu D, et al (2013) Influence of efavirenz pharmacokinetics and pharmacogenetics on neuropsychological disorders in Ugandan HIV-positive patients with or without tuberculosis: a prospective cohort study. BMC Infect Dis 13: 261. 23734829 10.1186/1471-2334-13-261 PMC3680019

[11] Elens L, Vandercam B, Yombi JC, Lison D, Wallemacq P, et al (2010) Influence of host genetic factors on efavirenz plasma and intracellular pharmacokinetics in HIV-1-infected patients. Pharmacogenomics 11: 1223–1234. 20860463 10.2217/pgs.10.94

[12] Faucette SR, Wang H, Hamilton GA, Jolley SL, Gilbert D, et al (2004) Regulation of CYP2B6 in primary human hepatocytes by prototypical inducers. Drug Metab Dispos 32: 348–358. 14977870 10.1124/dmd.32.3.348

[13] Faucette SR, Zhang TC, Moore R, Sueyoshi T, Omiecinski CJ, et al (2007) Relative activation of human pregnane X receptor versus constitutive androstane receptor defines distinct classes of CYP2B6 and CYP3A4 inducers. J Pharmacol Exp Ther 320: 72–80. 17041008 10.1124/jpet.106.112136 PMC4091905

[14] Habtewold A, Amogne W, Makonnen E, Yimer G, Nylen H, et al.. (2012) Pharmacogenetic and pharmacokinetic aspects of CYP3A induction by efavirenz in HIV patients. Pharmacogenomics J. 10.1038/tpj.2012.46 23089673

[15] Stohr W, Back D, Dunn D, Sabin C, Winston A, et al (2008) Factors influencing efavirenz and nevirapine plasma concentration: effect of ethnicity, weight and co-medication. Antivir Ther 13: 675–685. 18771051

[16] Burger D, van der Heiden I, la Porte C, van der Ende M, Groeneveld P, et al (2006) Interpatient variability in the pharmacokinetics of the HIV non-nucleoside reverse transcriptase inhibitor efavirenz: the effect of gender, race, and CYP2B6 polymorphism. Br J Clin Pharmacol 61: 148–154. 16433869 10.1111/j.1365-2125.2005.02536.x PMC1885008

[17] Hodder S, Arasteh K, De Wet J, Gathe J, Gold J, et al (2012) Effect of gender and race on the week 48 findings in treatment-naive, HIV-1-infected patients enrolled in the randomized, phase III trials ECHO and THRIVE. HIV Med 13: 406–415. 22416849 10.1111/j.1468-1293.2012.00991.x

[18] Schackman BR, Ribaudo HJ, Krambrink A, Hughes V, Kuritzkes DR, et al (2007) Racial differences in virologic failure associated with adherence and quality of life on efavirenz-containing regimens for initial HIV therapy: results of ACTG A5095. J Acquir Immune Defic Syndr 46: 547–554. 18193496 10.1097/qai.0b013e31815ac499

[19] Yimer G, Amogne W, Habtewold A, Makonnen E, Ueda N, et al (2012) High plasma efavirenz level and CYP2B6*6 are associated with efavirenz-based HAART-induced liver injury in the treatment of naive HIV patients from Ethiopia: a prospective cohort study. Pharmacogenomics J 12: 499–506. 21862974 10.1038/tpj.2011.34

[20] Yimer G, Ueda N, Habtewold A, Amogne W, Suda A, et al (2011) Pharmacogenetic & Pharmacokinetic Biomarker for Efavirenz Based ARV and Rifampicin Based Anti-TB Drug Induced Liver Injury in TB-HIV Infected Patients. PLoS One 6: e27810. 22162992 10.1371/journal.pone.0027810 PMC3232196

[21] Mugusi S, Ngaimisi E, Janabi M, Minzi O, Bakari M, et al (2012) Liver Enzyme Abnormalities and Associated Risk Factors in HIV Patients on Efavirenz-Based HAART with or without Tuberculosis Co-Infection in Tanzania. PLoS One 7: e40180. 22808112 10.1371/journal.pone.0040180 PMC3394799

[22] Gounden V, van Niekerk C, Snyman T, George JA (2010) Presence of the CYP2B6 516G> T polymorphism, increased plasma Efavirenz concentrations and early neuropsychiatric side effects in South African HIV-infected patients. AIDS Res Ther 7: 32. 20723261 10.1186/1742-6405-7-32 PMC2933581

[23] Aklillu E, Mugusi S, Ngaimisi E, Hoffmann MM, Konig S, et al (2011) Frequency of the SLCO1B1 388A>G and the 521T>C polymorphism in Tanzania genotyped by a new LightCycler(R)-based method. Eur J Clin Pharmacol 67: 1139–1145. 21630030 10.1007/s00228-011-1065-9

[24] Lopez-Cortes LF, Ruiz-Valderas R, Marin-Niebla A, Pascual-Carrasco R, Rodriguez-Diez M, et al (2005) Therapeutic drug monitoring of efavirenz: trough levels cannot be estimated on the basis of earlier plasma determinations. J Acquir Immune Defic Syndr 39: 551–556. 16044006

[25] Burhenne J, Matthee AK, Pasakova I, Roder C, Heinrich T, et al (2010) No evidence for induction of ABC transporters in peripheral blood mononuclear cells in humans after 14 days of efavirenz treatment. Antimicrob Agents Chemother 54: 4185–4191. 20660679 10.1128/AAC.00283-10 PMC2944620

[26] Mugusi SF, Ngaimisi E, Janabi MY, Mugusi FM, Minzi OM, et al (2012) Risk factors for mortality among HIV-positive patients with and without active tuberculosis in Dar es Salaam, Tanzania. Antivir Ther 17: 265–274. 22293579 10.3851/IMP1956

[27] Aklillu E, Dandara C, Bertilsson L, Masimirembwa C (2007) Pharmacogenetics of Cytochrome P450s in African Populations: Clinical and Molecular Evolutionary Implications. In: Suarez-Kurtz G, ed. Pharmacogenomics in Admixed Populations. Austin, TX: Landes Bioscience, 99–119.

[28] Jamshidi Y, Moreton M, McKeown DA, Andrews S, Nithiyananthan T, et al (2010) Tribal ethnicity and CYP2B6 genetics in Ugandan and Zimbabwean populations in the UK: implications for efavirenz dosing in HIV infection. J Antimicrob Chemother 65: 2614–2619. 20952418 10.1093/jac/dkq369

[29] di Iulio J, Fayet A, Arab-Alameddine M, Rotger M, Lubomirov R, et al (2009) In vivo analysis of efavirenz metabolism in individuals with impaired CYP2A6 function. Pharmacogenet Genomics 19: 300–309. 19238117 10.1097/FPC.0b013e328328d577

[30] Maimbo M, Kiyotani K, Mushiroda T, Masimirembwa C, Nakamura Y (2012) CYP2B6 genotype is a strong predictor of systemic exposure to efavirenz in HIV-infected Zimbabweans. Eur J Clin Pharmacol 68: 267–271. 21901344 10.1007/s00228-011-1118-0

[31] Heil SG, van der Ende ME, Schenk PW, van der Heiden I, Lindemans J, et al (2012) Associations between ABCB1, CYP2A6, CYP2B6, CYP2D6, and CYP3A5 alleles in relation to efavirenz and nevirapine pharmacokinetics in HIV-infected individuals. Ther Drug Monit 34: 153–159. 22354160 10.1097/FTD.0b013e31824868f3

[32] Gebeyehu E, Engidawork E, Bijnsdorp A, Aminy A, Diczfalusy U, et al (2011) Sex and CYP3A5 genotype influence total CYP3A activity: high CYP3A activity and a unique distribution of CYP3A5 variant alleles in Ethiopians. Pharmacogenomics J 11: 130–137. 20231858 10.1038/tpj.2010.16

[33] Nyakutira C, Roshammar D, Chigutsa E, Chonzi P, Ashton M, et al (2008) High prevalence of the CYP2B6 516G–>T(*6) variant and effect on the population pharmacokinetics of efavirenz in HIV/AIDS outpatients in Zimbabwe. Eur J Clin Pharmacol 64: 357–365. 18057928 10.1007/s00228-007-0412-3

[34] Hardwick RJ, Amogne W, Mugusi S, Yimer G, Ngaimisi E, et al.. (2012) beta-defensin Genomic Copy Number Is Associated With HIV Load and Immune Reconstitution in Sub-Saharan Africans. J Infect Dis. 10.1093/infdis/jis448 22837491

[35] Lugada ES, Mermin J, Kaharuza F, Ulvestad E, Were W, et al (2004) Population-based hematologic and immunologic reference values for a healthy Ugandan population. Clin Diagn Lab Immunol 11: 29–34. 14715541 10.1128/CDLI.11.1.29-34.2004 PMC321349

[36] Tsegaye A, Messele T, Tilahun T, Hailu E, Sahlu T, et al (1999) Immunohematological reference ranges for adult Ethiopians. Clin Diagn Lab Immunol 6: 410–414. 10225845 10.1128/cdli.6.3.410-414.1999 PMC103732

[37] Kassu A, Tsegaye A, Petros B, Wolday D, Hailu E, et al (2001) Distribution of lymphocyte subsets in healthy human immunodeficiency virus-negative adult Ethiopians from two geographic locales. Clin Diagn Lab Immunol 8: 1171–1176. 11687459 10.1128/CDLI.8.6.1171-1176.2001 PMC96245

[38] Saathoff E, Schneider P, Kleinfeldt V, Geis S, Haule D, et al (2008) Laboratory reference values for healthy adults from southern Tanzania. Trop Med Int Health 13: 612–625. 18331386 10.1111/j.1365-3156.2008.02047.x

